# An Account of the Practice of One of the Physicians of the Westminster General Dispensary, and of the Western Dispensary, from the 20th of December, 1807, to the 20th of January, 1808

**Published:** 1808-02

**Authors:** Samuel Fothergill

**Affiliations:** Southampton Street, Strand


*An Account of the Practice of one of the Physicians of the Westminster General Dispensary, and of the Western Dispensary, from the 20th of December,* 1807, *to the 20th of January,* 1808.

| | | | |
|---|---|---|---|
| ACUTE DISEASES. | | Inflammatory sore Throat | 2 |
| Catarrh - - | 8 | Fever - - | 3 |
| Peripneumony - | 2 | Measles - - | 4 |
| Pleurisy - - | 1 | Hooping-Cough - | 2 |
| Acute Rheumatism - | 4 | Acute Diseases of Infants | 10 |
| Inflammation of the Liver | 1 | | |

CHRONIC

| CHRONIC DISEASES. | |
|---|---|
| Cough and Dyspnœa | 28 |
| Pulmonary Consumption | 4 |
| Hæmoptoe - | 2 |
| Pleurodyne - - | 3 |
| Chronic Rheumatism | 8 |
| Lumbago and Sciatica | 2 |
| Cephalalgia - - | 3 |
| Tic Douloureux - | 1 |
| Gastrodynia - - | 4 |
| Enterodynia - - | 2 |
| Colica Pictonum - | 1 |
| Asthenia - - | 15 |
| Dyspepsia - - | 3 |
| Jaundice - - | 2 |
| Bilious Vomiting - | 2 |
| Dropsy - - - | 3 |
| Diarrhœa - - | 4 |
| Worms - - - | 3 |
| Gravel and Dysure - | 2 |
| Insanity - - | 1 |
| Marasmus - - | 1 |
| Debility and Cough after Measles - - - | 5 |
| Cutaneous Diseases - | 6 |
| Chlorosis - - - | 4 |
| Amenorrhœa - - | 3 |
| Menorrhagia - - | 2 |
| Leucorrhœa - - | 4 |

At this season of the year, pulmonic and rheumatic affections are the most frequent, as well as the most obstinate complaints which the Dispensary-Physician has to encounter. Many people are subject to them for several successive winters, and only enjoy intervals of remission from them during the mild weather, with which we are occasionally visited. The winter cough and dyspnœa, though severe and long continued, much seldomer terminate in consumption than might be apprehended. I have generally found that a combination of squills, opium, and ipecacuanha, has afforded relief, and in many recent instances speedily removed the complaints. Blisters, though in some cases of this nature eminently useful, much more frequently disappoint expectation; some patients, however, will have them; and as they seldom do harm, there cannot be much objection to their application. I have seen, however, some very unpleasant, and very dangerous consequences after their application to young children; a deep and spreading ulceration, very difficult to check, and sometimes assuming a gangrenous aspect, has taken place, and in one instance mortification occurred and proved fatal.

The measles, which have so long and generally prevailed, have now nearly disappeared; but have left numbers of children still affected with debility, cough, emaciation, and œdematous swellings of the face and extremities, which are very difficult to remove.

In three cases of catarrh, attended by pain in the side and chest, which was much increased by a frequent cough and shortness of breath, I directed from 12 to 16 ounces of blood blood to be taken from the arm; and was much pleased to observe the symptoms were immediately relieved, and the patients recovered sooner than I believe they would have done, had the lancet not been employed; they were strong men in the prime of life, accustomed to a full diet, and I attended them very early in the complaint. In such cases bleeding is strongly insisted upon by Sydenham, whilst the experience of modern physicians is against it. But the judicious practitioner regards not authority alone, nor prescribes to the name of a disease; he considers the urgency of the symptoms, the time that has intervened since their first appearance, and the nature of his patient's constitution.

The Dispensary-Physician enjoys admirable opportunities of seeing disease in every stage of its progress; he not unfrequently attends his patient on the second or third day of his attack, which in all febrile complaints is of the highest importance, for very formidable approaches of disease will occasionally yield to vigorous remedies when early employed; and though we fail in our first object, we may perhaps succeed in rendering the disorder less severe. In three or four instances, I have lately had reason to suppose that I was successful in checking the accession of continued fever and acute rheumatism, when I was called in on the second day of the attack. When these complaints are firmly established in the system, and a certain degree of febrile action is excited, we well know that medicine will neither shorten their duration, nor secure the patient from danger; we may indeed sometimes mitigate the violence of symptoms, obviate the mischievous effects of busy interference, and in some degree beneficially direct the efforts of nature; but the disease will go on, and if we save the patient, it is not seldom by doing nothing, which in many cases is doing every thing, for it gives a fair chance of recovery. By earnest attention to the case, we infuse confidence into the mind of the patient, keeping up his spirits by the cheerfulness of hope, and thus apparently wonderful cures are accomplished by the simplest means.

SAMUEL FOTHERGILL.

*Southampton Street, Strand, Jan. 22,* 1808.

Account

